# Growth-Defense Trade-Offs Induced by Long-term Overgrazing Could Act as a Stress Memory

**DOI:** 10.3389/fpls.2022.917354

**Published:** 2022-06-02

**Authors:** Kairi Qu, Yunxiang Cheng, Kairu Gao, Weibo Ren, Ellen L. Fry, Jingjing Yin, Yaling Liu

**Affiliations:** ^1^School of Ecology and Environment, Inner Mongolia University, Hohhot, China; ^2^Department of Biology, Edge Hill University, Ormskirk, United Kingdom; ^3^Inner Mongolia Mongolian Grass Seed Industry Science and Technology Research Institute Co., Ltd., Hohhot, China

**Keywords:** phytohormone, stress memory, overgrazing, *Leymus chinensis*, growth-defense

## Abstract

Long-term overgrazing (OG) is one of the key drivers of global grassland degradation with severe loss of productivity and ecosystem functions, which may result in stress memory such as smaller stature of grassland plants. However, how the OG-induced stress memory could be regulated by phytohormones is unknown. In this study, we investigated the changes of four phytohormones of cloned offspring of *Leymus chinensis* that were developed from no-grazing (NG) plants and OG plants with a grazing history of 30 years. The concentrations of auxin (IAA) and gibberellic acid (GA) in OG plant leaves were 45% and 20% lower than control, respectively. Meanwhile, the level of abscisic acid (ABA) in OG leaves nearly doubled compared with that in NG leaves. The situation was quite similar in roots. Unexpectedly, no significant changes in the jasmonic acid (JA) level were observed between OG and NG plants. The changes in gene expression patterns between OG and NG plants were also investigated by transcriptomic analysis. In total, 302 differentially expressed genes (DEGs) were identified between OG and NG plants, which were mainly classified into the functions of synthesis, receptor, and signal transduction processes of phytohormones. The expression of 24 key genes related to the biosynthesis and signal transduction of IAA and GA was downregulated in OG plants. Among them, *OASA1* and *AO1* (regulating the biosynthesis of IAA and ABA, respectively) were reduced significantly by 88 and 92%, respectively. In addition, the content of secondary metabolites related to plant defense such as flavonoids and phenols was also increased in leaves. Taken together, the decrease of positive plant growth-related hormones (IAA and GA) together with the increase of plant stress-related hormones or factors (ABA, flavonoids, and phenols) induced the growth-defense trade-offs for *L. chinensis* adaptation to long-term OG stress. The findings reported in this study shed new light on the mechanism of plant–animal interaction in the grassland ecosystem and provide a deeper insight into optimizing grazing management and sustainable utilization of grassland.

## Introduction

Serving as a global reservoir of biodiversity and a provider of direct and indirect benefits to humans, grassland is one of the most important terrestrial ecosystems, covering 40% of the Earth's surface (Bardgett et al., 2021). However, it is subject to widespread degradation, with overgrazing (OG) as one of the key drivers, inducing desertification (Ibáñez et al., 2007). In the 2000s, approximately 90% of grassland had been degraded to some extent in China (Liu et al., 2019), which can be characterized by a decrease in grassland productivity. Recently, it has been suggested that long-term OG can lead to stunting of grassland plants over multiple generations, and this dwarfism could persist even after stress was removed (Li et al., 2015).

*Leymus chinensis*, a perennial rhizome plant, is the dominant and most important fodder species of the typical steppe. Reports show a consistent trait shift including dwarfed height, shorter and narrower leaves, smaller cluster width, and shallow root distribution under long-term OG (Wang, 2004). Stress memory, when plants store and retain information of previous stress cues and exhibit a much stronger and faster response to recurring events, is regulated by different mechanisms (Saqlain et al., 2021). Stress such as drought, cold, and insect invasion can induce stress memory which can help plants cope with these stresses (Rasmann et al., 2012; Walter et al., 2013; Mantoan et al., 2020). Many phytohormones such as ABA and JA were found to play a key role in the maintenance of stress memory (Avramova, 2019). Recent reports showed that long-term OG could also produce stress memory such as smaller plant size and leaves and increased secondary metabolites such as flavonoids (Ren et al., 2017; Liu et al., 2019a). However, stress memory can help plants defend against animal grazing, but how this grazing-induced stress memory works still unclear. We have found that there was stress memory induced by long-term OG in the offspring of *L. chinensis* (Ren et al., 2017), but the regulatory mechanism is still being explored. Phytohormones are characterized as messengers, which act to regulate different plant traits (Koepfli et al., 1938; Sharma et al., 2021). Recent studies have shown that abscisic acid (ABA), auxin (IAA), gibberellin (GA), cytokinin (CTK), jasmonic acid (JA), salicylic acid (SA), brassinosteroids (BR), and peptide hormones are involved in plant defense signaling pathways, which are likely to be key agents in response to OG (Bari and Jones, 2009; Urano et al., 2017; Tzipilevich and Benfey, 2021). Hormones not only respond to abiotic stress but also contribute greatly to mitigate biotic stress. For biotic stress, ABA and JA pathways synergistically mediate responses of defense-related genes to repeated herbivore stresses (Avramova, 2019). These changes may influence the responses of plants to recurring stress and form a memory to cope with the next threat. We expect that long-term OG also causes *L. chinensis* to develop stress memory which is expressed as dwarfed plants with smaller leaves, as a result of upregulation of specific phytohormones such as ABA or JA. Additionally, OG stress in the wild can lead to the activation of some genes in ABA and JA signaling pathways in *L. chinensis*, thus enhancing its defense ability through modulating metabolism and development such as stomatal closure and secondary metabolite content (Zhang et al., 2020).

Dwarf forms of long-term grazed *L. chinensis* may be an adaptation to long-term grazing by large herbivores (Li et al., 2015). At the transcriptome and proteome level, the expression of the genes involved in defense and immune responses, pathogenic resistance, and cell development was changed, which collectively inhibited the growth of clonal *L. chinensis* (Ren et al., 2018a). Furthermore, the proteins associated with dwarfism induced by OG were also altered, including upregulated ATPB_DIOEL and downregulated DNAK_GRATL, as well as proteins that interact with them, such as RPOB2_LEPTE, A0A023H9M8_9STRA, and RBL_AMOTI, (Ren et al., 2018b). Interestingly, it was found that clonal transgenerational effects in *L. chinensis* phenotypic traits heavily involve photosynthetic plasticity (Ren et al., 2017). Phytohormones such as IAA can regulate growth and development by controlling photosynthesis (Malkowski et al., 2020). Do phytohormones also play an important role in dwarf forms induced by long-term OG?

The aim of this research was to explore how phytohormones changed and regulated after plants experienced long-term OG. We questioned the following: (1) Which phytohormones related to growth and defense pathways play roles in the maintenance of stress memory induced by long-term OG? (2) How are these phytohormones regulated by key genes? (3) How does the downstream defense system of secondary metabolites respond? The findings will expand our knowledge of the mechanism of how plants respond to long-term OG.

## Materials and Methods

### Field Site and Plant Material

Our experimental materials were taken from Baiyinxile Pasture in Xilingol League, Inner Mongolia which belongs to the Inner Mongolia Grassland Ecosystem Research Station (IMGERS, 43°38′N, 116°42′E, 1,211 m). The research site is in a northern temperate semiarid climate, with an average annual temperature of 0.3°C (lowest monthly average temperature of −21.6°C in January and the highest of 19.0°C in July) and annual precipitation of 270.78 mm. Precipitation is concentrated from May to September when the temperature and moisture are best for plant growth. Dominant vegetation species are *L. chinensis* and *Stipa grandis* (Ren et al., 2017).

We sampled from two plots: one grazing exclosure plot that has been fenced since 1983, while the outside of the exclosure has been subjected to long-term OG, with a stocking rate of three sheep units per hectare for more than 50 years. We collected rhizomes of *L. chinensis* from the exclosure plot (no grazing, NG) and outside of the exclosure plot (long-term OG) in September 2020. In each plot, six lines were set parallel to the fence, and a point was set every 20 m on each plotline to collect five samples of *L. chinensis* rhizomes. To avoid cross-area interference between the NG and OG plots, all lines were more than 30 m away from the fence. Then we brought them back for potted culture in the greenhouse.

### Greenhouse Experiment

We replanted rhizome buds selected randomly from NG and OG individuals before the start of the *L. chinensis* growing season, in May 2021. Different parent plants were cut to 2–3 cm lengths and each rhizome with a bud, and then the materials were planted into 18 cm depth pots after the pots were filled with nutritional soil. Each treatment (NG and OG) was replicated 30 times across a total of 60 pots (2 treatments × 30 replicates). The average temperature was 25°C during the day and 15°C at night. Samples were measured when the seedlings had grown for 6 weeks.

### Morphological Traits

We measured the morphological traits of *L. chinensis* at harvest including height, leaf number, tiller number, leaf width, leaf length, leaf area, aboveground biomass, belowground biomass, and total biomass. The height was measured as the vertical height of the plant. Leaf morphological traits were measured using the second leaf from the top of the materials using a leaf area meter (LI-3000 Portable Area meter Produced by LI-COR Lincoln, NE, USA). For the biomass, we divided the individuals into aboveground and belowground, then cleaned and dried them at 65°C for 24 h, and weighed them to 0.01 accuracy.

### Phytohormone Content

Phytohormone analyses were carried out according to Yilamujiang et al. (2016) with some modifications. Plants were separated into leaves and roots and then the tissue samples were ground to powder with liquid nitrogen. After grinding, the samples were put into a 2 ml centrifuge tube, followed by adding methanol-acetonitrile-aqueous solution (40:40:20, v/v), shaken, mixed for 2 min, and then extracted at 4°C for 12 h under light protection, and centrifuged at 14,000 r/min for 10 min. The supernatant was taken and dried with nitrogen. Methanol-aqueous solution (50:50, v/v) was centrifuged at 14,000 r/min for 10 min at constant volume, and the supernatant was taken for sample analysis.

The chromatographic conditions were as follows: mobile phase, liquid A was 0.04% formic acid-aqueous solution, liquid B was 0.04% formic acid-acetonitrile solution, column temperature was 45°C, the flow rate was 400 μl, min^−1^, and sample volume was 4 μl. Chromatographic column, Waters, ACQUITY UPLCBEHC18 (2.1 mm × 100 mm, I.D. 1.7 μm); electrospray electric ion source (ESI), ion source temperature 500°C. The detection equipment was the Agilent 1290 HPLC-MS System.

### Secondary Metabolite Content

The roots and leaves of the plant samples were separated prior to the quantification of secondary metabolites. The tissue samples were dried to a constant weight, crushed, and sifted through 40 mesh.

#### Tannins

Tannin content was determined based on the study by Kabir et al. (2015). Briefly, the volume of distilled water (ml) was 1:5-10 (approximately 0.1 g of tissue was weighed and 1 ml of distilled water was added). After full homogenization, the liquid was transferred to an EP tube, extracted in a water bath at 80°C for 30 min and centrifuged at 8,000 *g* at 25°C for 10 min. The supernatant was taken and tested. Notably, 0.5 ml of supernatant was mixed with 3 ml of 4% vanillin methanol solution and 1.5 ml of pure hydrochloric acid, and the absorbance was measured at 510 nm using a microplate reader (Multiskan GO 1510, Thermo). Tannin content was measured using the standard curve of the spectrometer.

#### Total Phenols

Approximately 0.1 g of each sample was weighed, 2.5 ml of 60% ethanol was added, and then shaken for 2 h at 60°C. The supernatant was centrifuged at 10,000 *g* for 10 min at 25°C, and the volume of the extract to be tested was fixed to 2.5 ml. Of note, 0.5 ml of supernatant was mixed with 5 ml of distilled water and 0.5 ml of Folin-Ciocalteu reagent (Al-Saeedi and Hossain, 2015). Notably, 2 ml of 20% Na_2_CO_3_ was then added, and the mixture was kept in water at 50°C for 30 min. The absorbance was measured at 750 nm using a microplate reader (Multiskan GO 1510, Thermo).

#### Flavonoids

Flavonoids were measured according to the aluminum nitrate colorimetric method (Xiang N. et al., 2021). Approximately 0.02 g was weighed, and 2 ml of the anhydrous ethanol was added. The sample was shaken for 2 h at 60°C and then centrifuged for 10 min at 25°C. Notably, 1 ml of supernatant was mixed with 1 ml of 70% methanol and 0.3 ml of 5% sodium nitrate, stored at room temperature for 6 min, and then 0.3 ml of aluminum nitrate was added to each tube. After 6 min, 3 ml of 4% sodium hydroxide was added to dissolve the mixed solution. The absorbance of the solution was measured at 510 nm using a microplate reader (Multiskan GO 1510, Thermo). The established standard curve was used to calculate the content of total flavonoids in the samples.

### RNA Extraction

RNA was extracted from roots and leaves separately using the factory protocol (TIANGEN Company, DP419 total RNA Extraction Kit). RNA purity, concentration, and integrity were measured using spectrophotometers (NanoDrop, Qubit 2.0, and Agilent 2100).

### Transcriptome Sequencing

The samples were from the RNA extraction. The eukaryotic mRNA with a polyA tail was enriched by magnetic beads with Oligo(dT), and the mRNA was interrupted by ultrasound. The first cDNA strand was synthesized in the m-Mulv reverse transcriptase system using fragment mRNA as a template and random oligonucleotide as a primer. Subsequently, the RNA strand was degraded by RNaseH, and the second cDNA strand was synthesized by dNTPs in the DNA Polymerase I system. The purified double-stranded cDNA was repaired at the end, an A tail was added, and sequencing joints were connected. Approximately 200 bp cDNA was screened with AMPure XP Beads for PCR amplification. The PCR products were purified again with AMPure XP Beads to obtain the library. Analysis was carried out using the EdgeR and DESeq2 software, including standardization of read counts and calculation of *P* and FDR values.

### Quantitative Real-Time PCR Analysis

National Center for Biotechnology Information (https://www.ncbi.nlm.nih.gov) was used to design primer sequences. The corresponding real-time PCR primers (Supplementary Table S1) were designed using the sequence information obtained by sequencing, and then the extracted RNA was reverse-transcribed into cDNA, which was diluted to a certain multiple according to its concentration. A real-time PCR reaction was performed on an ABI real-time PCR machine (QuantStudio 3, Thermofisher), and each cDNA sample was repeated three times. ΔCT = CTtreatment-CTreference and ΔΔCT= ΔCTgrazing-ΔCTnograzing relative to normal grazing were calculated for relative gene expression differences. After internal homogenization, the relative expression differences of target genes were expressed as a 2-ΔΔCT value, indicating the differential expression multiple of overgrazed *L. chinensis* relative to enclosure *L. chinensis*.

### Statistical Analysis

We reported the results as means ± SE for all the above indicators. The SPSS statistical software version 26.0 (SPSS, Chicago, Illinois, USA) was used for ANOVA, significant difference (*P* < 0.05) detection, and graphical representations. Read count data obtained from the gene expression level analysis were analyzed using the edgeR and Deseq2 software, including the standardization of read counts and calculation of *P* and FDR values. Statistical test FDR value and differential multiple log2FC were used to screen differential genes. The default threshold was FDR < 0.05.

## Results

### The Phenotype of Grazing Plants Showed Dwarfism

**Figure 1:**
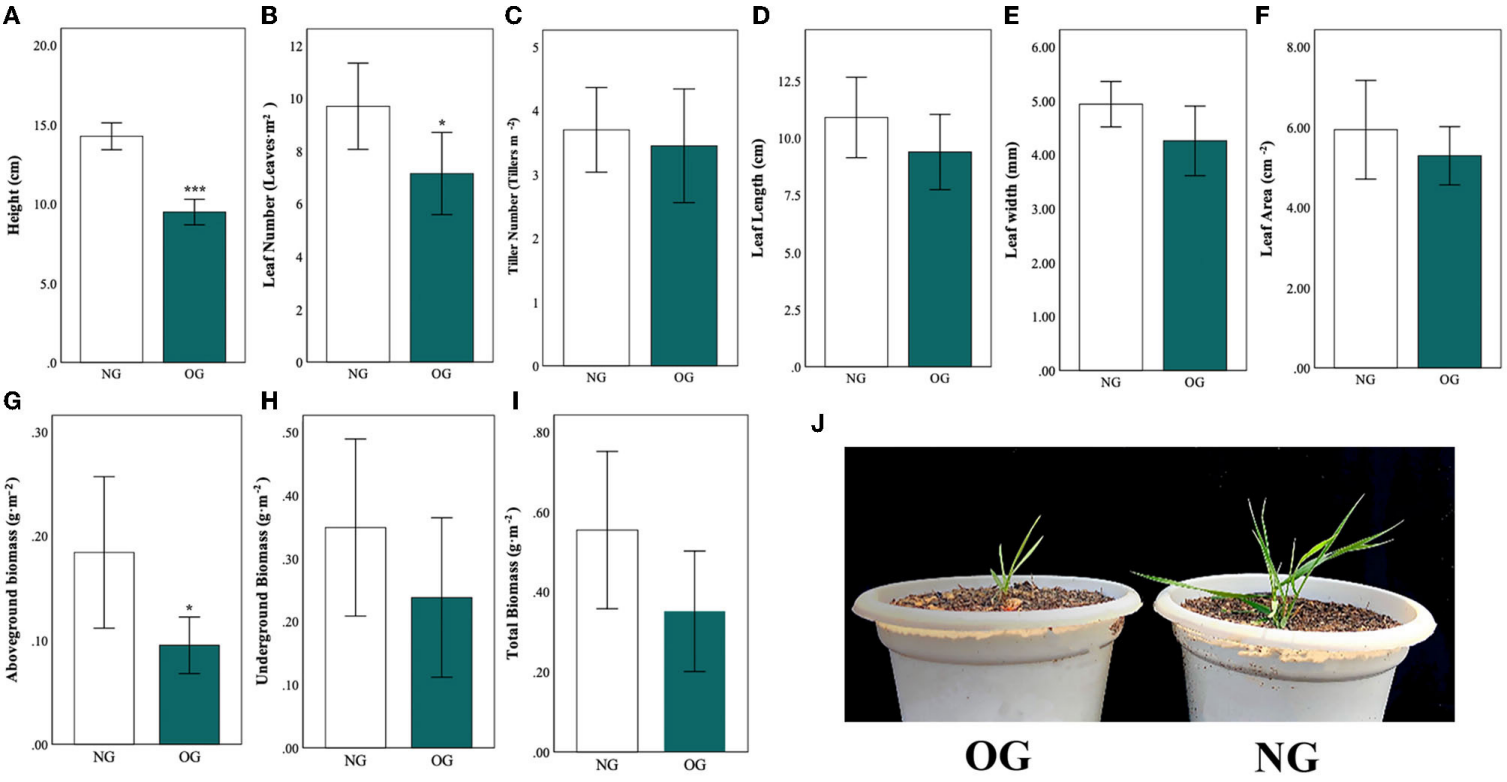
Differences in plant morphological traits in *Leymus chinensis* in response to no-grazing (NG) and overgrazing (OG) conditions. **(A–I)** show different morphological traits, **(J)** comparison of early growth stage. Statistical significance is denoted by ***P < 0.001; **P < 0.01; *P < 0.05. Error bars denote standard error of the mean.

All measured morphological traits were reduced in OG plants compared with NG (Figure 1). Three traits were significantly lower (Figure 1A: plant height, Figure 1B: leaf number, and Figure 1G: aboveground biomass), with the height of the OG plants on average 50% lower than that of the NG plants (*P* < 0.001). Compared with the NG control, the leaf number of the OG plant decreased by approximately 30% (*P* = 0.023), while the aboveground biomass of OG plants was only half as much as the NG plants (*P* = 0.018). Besides, other trait as tiller number (Figure 1C), leaf length (Figure 1D), leaf width (Figure 1E), leaf area (Figure 1F), underground biomass (Figure 1H) and total biomass (Figure 1I) reduced with no significance.

### Hormone Content Response to Grazing

**Figure 2:**
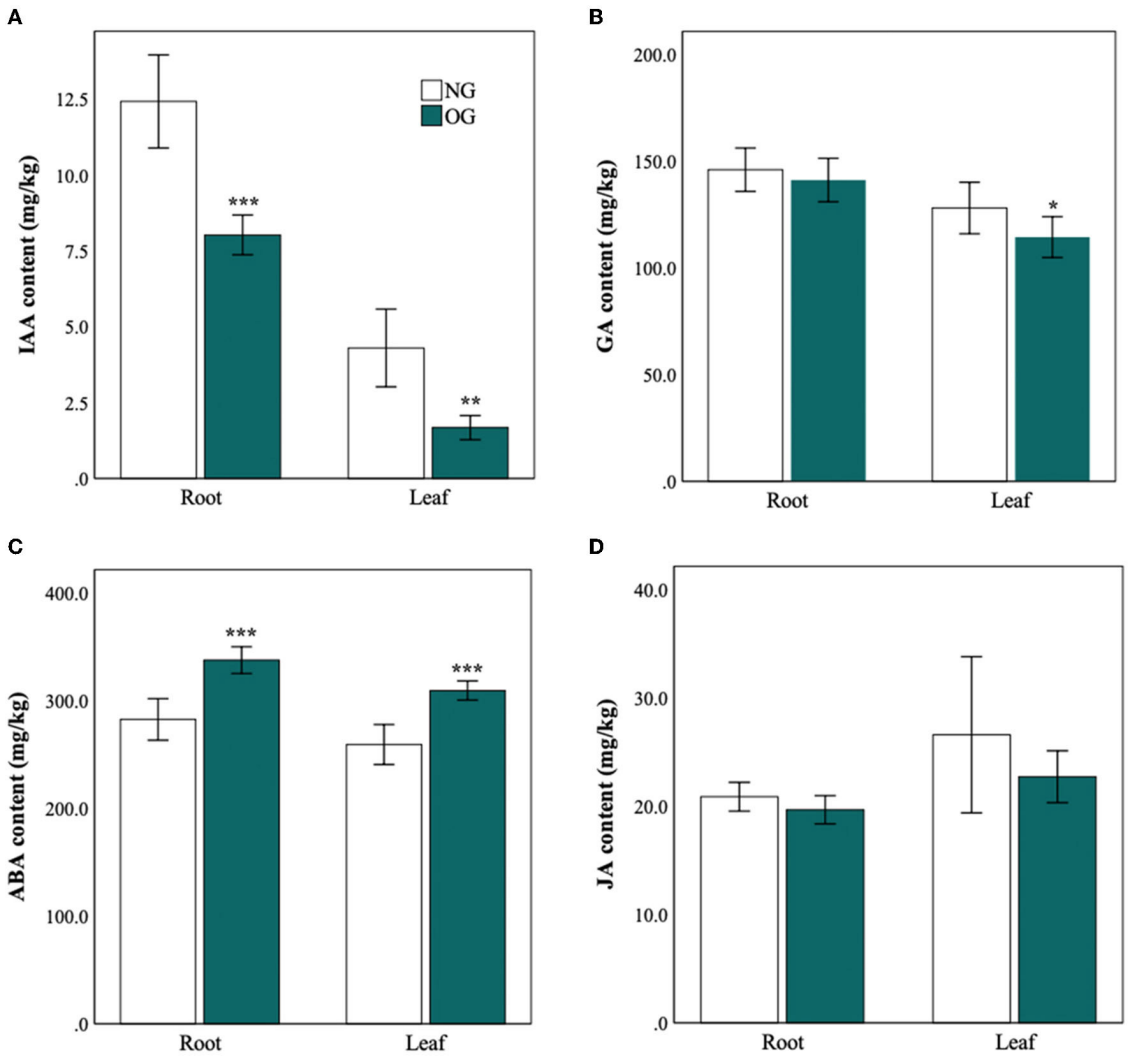
Different hormone levels in roots and leaves of *L. chinensis* in response to NG and OG. **(A)** Auxin (IAA) concentration; **(B)** gibberellic acid (GA) concentration; **(C)** abscisic acid (ABA) concentration; and **(D)** jasmonic acid (JA) concentration. Statistical significance is denoted by ***P < 0.001; **P < 0.01; *P < 0.05. Error bars denote standard error of the mean.

The IAA concentration was significantly reduced in both roots and leaves in the OG plants compared with the NG (Figure 2A). GA was also lower in the leaves of *L. chinensis*, but there was no significant effect of grazing on GA in the roots (Figure 2B). Moreover, the concentration of IAA and GA in leaves and roots also decreased after plants experienced long-term OG (Figures 2A,B). Compared with NG plants, the content of IAA in OG plants reduced to 45% (*P* < 0.001) in leaves and 65% in the roots (*P* = 0.001). As to GA, there was a 20% reduction in OG leaves (*P* = 0.03). The situation of ABA was quite different. The content of ABA of OG plants increased 1.2-fold in roots and 1.1-fold in leaves (*P* < 0.001) (Figure 2C). The content of JA in OG plants shows slight decreases relative to that of NG plants, but with no significant difference (Figure 2D).

### Analysis of Hormone Biosynthesis or Metabolism-Related Genes Under OG Stress Memory

**Figure 3:**
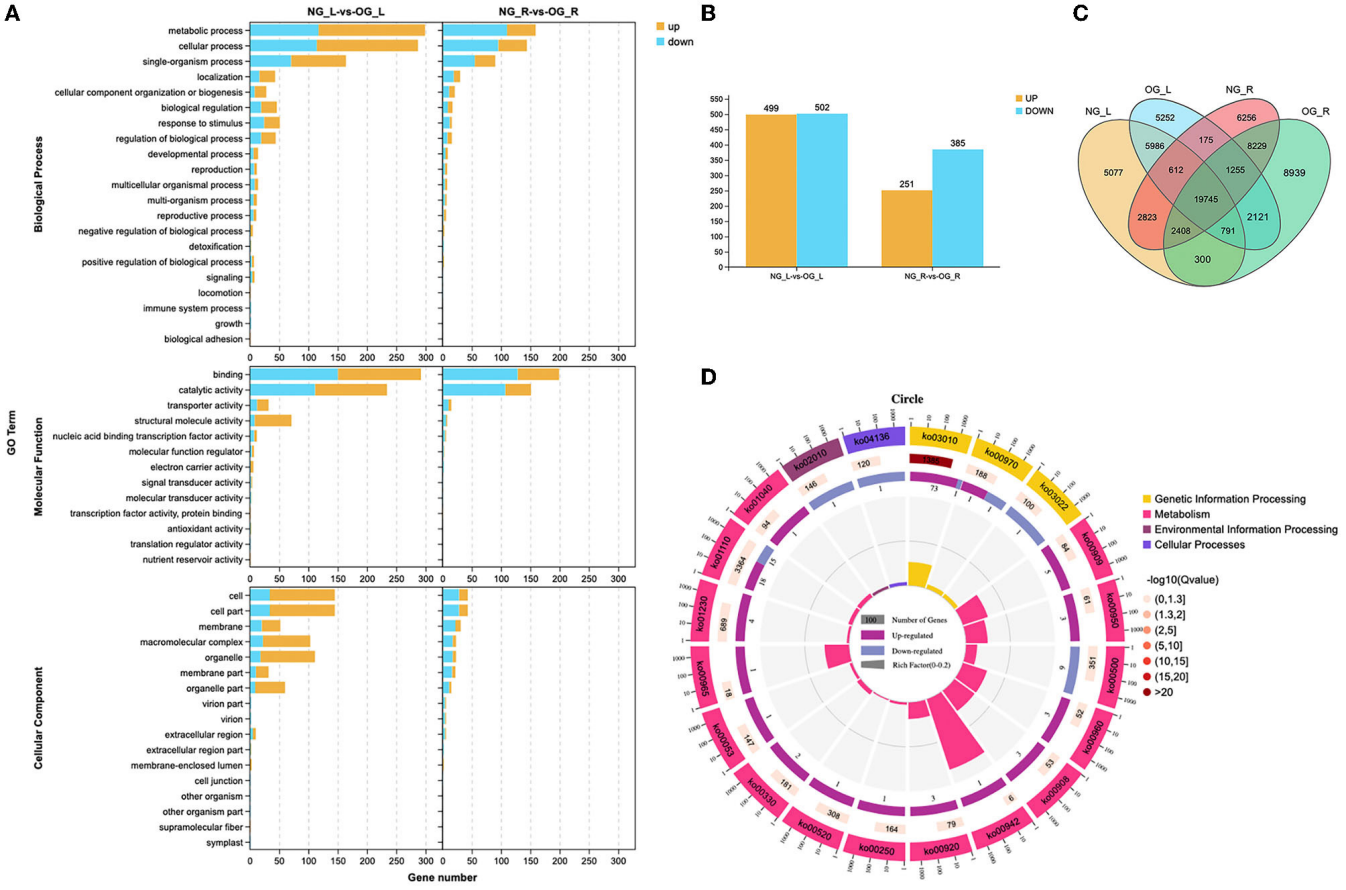
Differences between the NG and OG plants at the transcriptome level. **(A)** Gene ontology (GO) enrichment maps of differentially expressed genes (DEGs). **(B)** Number of DEGs between NG and OG plants. **(C)** Venn diagram of all genes between the leaf and root. **(D)** Kyoto Encyclopedia of Genes and Genomes (KEGG) enrichment maps of DEGs. NG_L, leaf of no-grazing plants; NG_R, root of no-grazing plants; OG_L, leaf of overgrazing plants; OG_R, root of overgrazing plants.

There were 1,001 differentially expressed genes (DEGs) in leaves, where 499 were upregulated and 502 were downregulated (Figure 3B). There were 636 DEGs in the roots, 251 of which were upregulated and 385 were downregulated. In total, 1,646 DEGs were found between the leaf and root (Figure 3C), which were enriched in metabolism, environmental information processing, and genetic information processing based on gene ontology (GO) enrichment analysis (Figure 3A). In addition, the Kyoto Encyclopedia of Genes and Genomes (KEGG) analysis indicated that most DEGs were enriched in genetic information processing, metabolism, environmental information processing, and cellular processing (Figure 3D).

**Figure 4:**
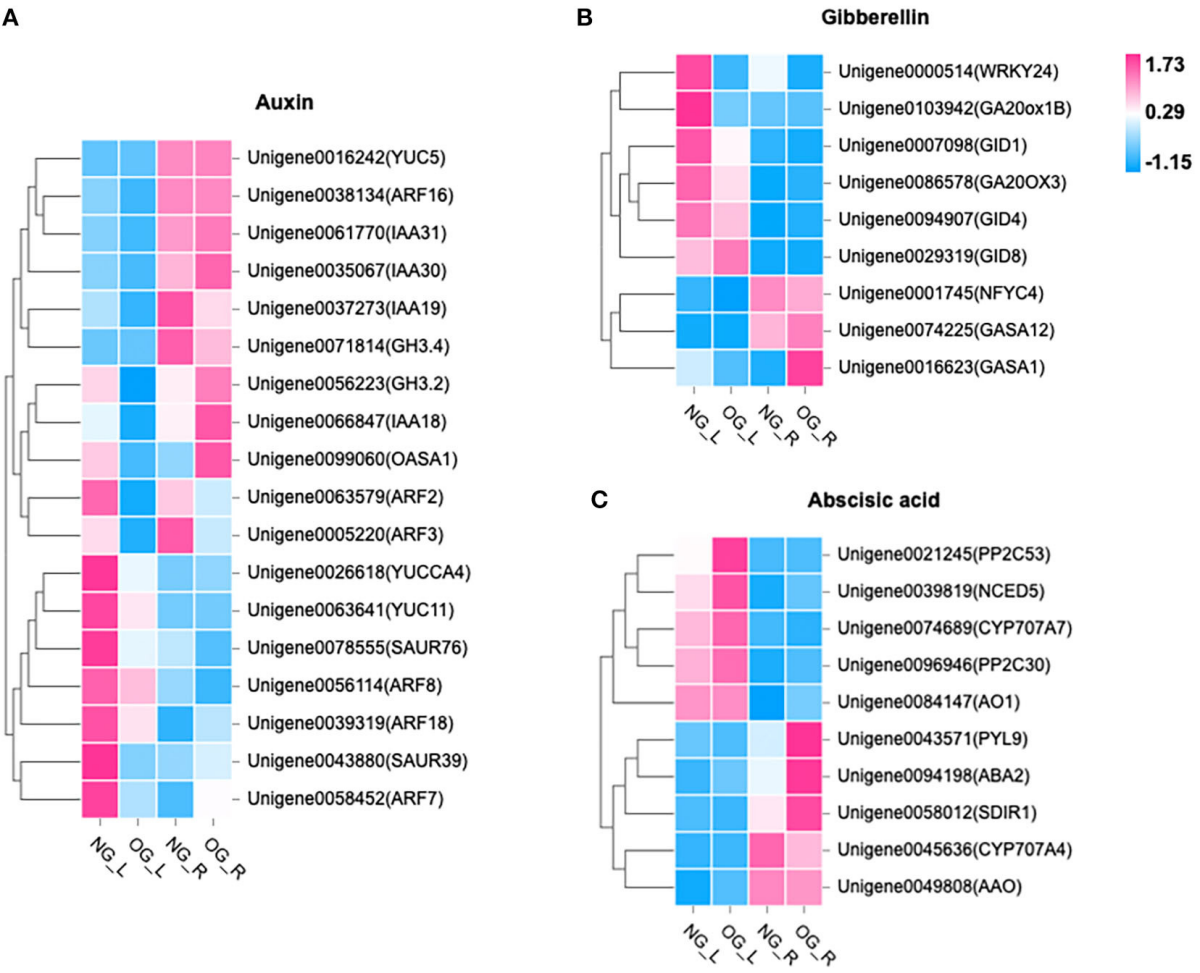
Changes in the expression of genes in response to long-term OG stress. The heat map shows the relative transcript levels of selected biosynthetic, metabolic, and signaling genes for **(A)** IAA, **(B)** ABA, and **(C)** GA. NG_L, leaf of no-grazing; NG_R, root of no-grazing; OG_L, leaf of overgrazing; OG_R, root of overgrazing.

Among the DEGs, 302 genes in leaves were identified to be related to biosynthesis pathways and signal transduction of auxin, ABA, and gibberellin. Compared with NG plants, the expression level of nine genes related to IAA biosynthesis and signal transduction was downregulated in OG plants (Figure 4A). The expression level of four genes related to GA biosynthesis and signal transduction was also downregulated in OG plants (Figure 4B). As to ABA, the situation was quite different. The expression level of four genes related to ABA biosynthesis and signal transduction was upregulated in OG plants compared with NG plants (Figure 4C).

Among them, six genes were selected, and their expression levels were verified by RT-PCR testing (Supplementary Figure S1). The expression of two genes related to IAA and GA biosynthesis and signal transduction was significantly downregulated by OG, among which the expression of *OASA1* reduced by 88% (*P* = 0.023) and *GID* lowered approximately 671% compared to NG (*P* = 0.017). Meanwhile, there was a 92% increase in expression of the ABA-related gene *AO1* (*P* < 0.05), while *PP2C53* rose five times (*P* < 0.05).

### Secondary Metabolite Concentration

**Figure 5:**
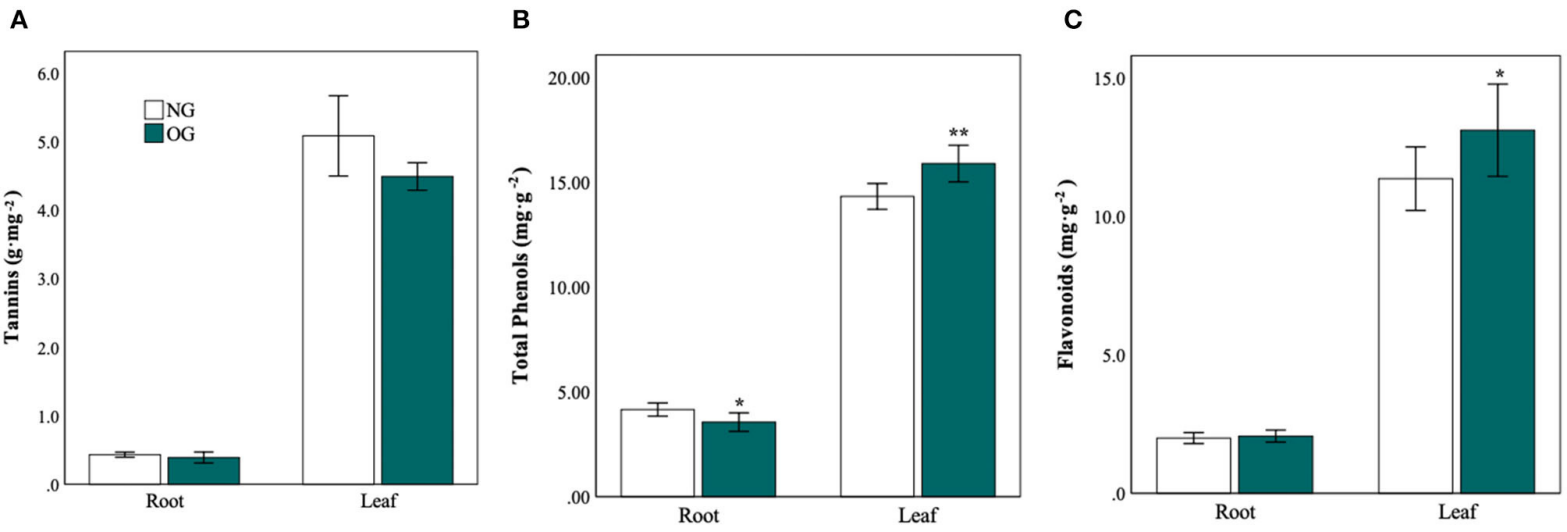
Plant defense compounds in roots and leaves in response to a legacy of OG metabolism. **(A)** Tannins, **(B)** flavonoids, and **(C)** total phenols in roots and leaves in response to two conditions. Statistical significance is denoted by ***P < 0.001; **P < 0.01; *P < 0.05. Error bars denote the standard error of the mean.

To further explore the changes in defense-related secondary metabolites, we measured the content of tannins (Figure 5A), total phenols (Figure 5B), and flavonoids (Figure 5C) in leaves and roots. As to the leaves, both the content of total phenols and flavonoids in OG plants increased 110% (*P* = 0.004) and 15% (*P* = 0.043) compared with NG plants. The content of tannins in OG plants decreased slightly than that in NG plants (*P* > 0.05). As to roots, the content of total phenols in OG plants decreased by approximately 17% (*P* = 0.026) compared with NG plants. Moreover, the activity of peroxidase (POD) and superoxide dismutase (SOD) in the roots was significantly reduced by 56% and 49%, respectively (Supplementary Figure S2).

## Discussion

### Plants of *L. Chinensis* Tend to Be Small Due to Stress Memory

As one of the main natural enemies of plants, herbivores can damage plants by chewing, trampling, and saliva deposition (Chen et al., 2021). Under such biotic stress, plants develop multiple defense mechanisms to address these stresses, and many of them can be passed on to their offspring. In this study, we found that plants tend to be much smaller with decreased plant height and aboveground biomass (Figure 1J). This finding is quite similar to previous reports (Li et al., 2015). This can be explained by plant grazing avoidance mechanisms (Suzuki and Suzuki, 2011) induced by stress memory, in which morphological plasticity of clones of grassland plants caused a significant reduction in plant biomass which directly led to the decline of grassland productivity (Li et al., 2014), in order to avoid herbivores.

### Stunting May Be Regulated by the Phytohormones IAA, GA, and ABA

In our study, ABA was found to play an important role in long-term grazing stress. In the meanwhile, plant growth-enhancing factors such as IAA and GA were also involved to cope with long-term OG stress. Contrary to our expectations, we did not find a significant effect of OG on JA concentration in root or leaf tissue. Previous reports indicated that JA can often be used as a signal to trigger herbivore-induced defense (Li et al., 2002; Koo and Howe, 2009; Qi et al., 2016; Hu et al., 2021). The content of JA in leaves was significantly higher in the extremely heavy grazing plot than that in the NG treatment under field conditions (Liu et al., 2019). One of the possible reasons is that plants may choose different phytohormone responses to OG with a different grazing history. As to short-term OG stress, JA is often used as a signal to activate the defense system against biotic stress (Liu et al., 2019). Some researchers have observed that the accumulation of JA isoleucine was one necessary condition for ABA synthesis under abiotic stress, so it can be postulated that JA regulation is upstream of ABA biosynthesis (Teng et al., 2014). In contrast, ABA can inhibit the expression of JA pathway-related genes, which suppress the resistance to pathogen infection mediated by JA (Xie et al., 2018). We considered that when exposed to long-term OG, plants tend to use ABA instead of JA as signals enhancing their tolerance. In total, the interaction of these hormones regulates the growth-defense trade-off to affect plant growth under grazing stress (Wang and Irving, 2011).

### Phytohormones Are Regulated at the Gene Level

To explore how these key phytohormones are regulated after long-term OG stress, the gene expression pattern between OG and NG was investigated. DEGs in the leaves were nearly doubled than that in the roots. This can be explained by the fact that the leaves were more sensitive to OG than roots. Then, we found genes involved in hormone synthesis, metabolism, and signal transduction in leaves of *L. chinensis*. The *YUC* family has been proposed as crucial genes that act in the common IAA biosynthetic pathway (Mashiguchi et al., 2011), and the anthranilate synthase (AS) gene encodes a key enzyme in the synthesis of tryptophan (Trp) (Tozawa, 2001). The expression levels of *YUC11, YUCCA4*, and *OASA1* were strongly downregulated (Figure 4A) which regulates auxin synthesis (Du et al., 2013). IAA plays an essential role in lateral organ initiation at the shoot apical meristem, patterning, and vascular development, maintaining stem cell fate at the root apical meristem, as well as promoting branching in the root (Wolters and Jurgens, 2009). This means that the content of IAA in the plant is restricted, which affects the growth of *L. chinensis*.

Similarly, several key genes that participated in the biosynthesis of GA including *GID1, GID4, GID8, GA20OX3*, and *GA20ox1B* were induced by OG stresses. *GID1* is a key gene of the GA receptor, and *G20ox* is a GA synthesis gene. GA is essential for determining plant height by regulating stem elongation (Zhang et al., 2016), so it is likely that GA is involved in the dwarfing of *L. chinensis*.

Additionally, genes such as *AO1, SDIR1, ABA2*, and *NCED5* related to ABA synthesis were significantly upregulated in OG plants. ABA helps plants respond to abiotic stress, especially in water-deficit responses (Vickers et al., 2009). In addition, ABA regulates the stomatal aperture by changing the volume of guard cells that control gas exchange (Chen et al., 2021). Our previous study found that stomatal conductance of overgrazed *L. chinensis* significantly decreased (Yin et al., 2020), which may be related to ABA content changes.

### The Secondary Metabolites of *L. Chinensis* Were Changed After Long-Term OG

Plants can protect themselves from biotic or abiotic stress through the synthesis of secondary metabolites. It has been revealed by phytochemical studies that the secondary metabolites contain alkaloids, terpenoids, flavonoids, and phenols (Mutuku et al., 2020). Under the wild condition, grazing can cause changes in the content of secondary metabolites (Liu et al., 2019b). The secondary metabolites, especially flavonoids, exist widely in plants and are involved in responses to biotic and abiotic stresses (Baozhu et al., 2022), which play the role of a defensive weapon. The change in hormone content may cause a difference in flavonoid synthesis (Nik and Durkin, 2018). For example, ABA has been used to induce the biosynthesis of secondary metabolites such as phenol, flavonoid, anthocyanin, and carotenoid production in *Dracocephalum moldavica L*. (Khaleghnezhad et al., 2019). Plants often enhanced their defense induced by animals or insects by the increase of some specific secondary metabolites such as alkaloids, flavanols, and phenols. Among them, many phenols were reported to inhibit herbivore growth and reproduction, even by direct toxicity (Huitu et al., 2014). Similarly, flavanols have digestibility-reducing effects in herbivores (Tohge et al., 2017), which is a relatively cheap defense due to its low biosynthetic costs (Scogings et al., 2021). Interestingly, in this research, GA content did not change significantly in roots, while the concentration of tannins and phenols declined. At the same time, the activity of POD and SOD in the roots was significantly reduced (Supplementary Figure S1), which may facilitate endophytic colonization by suppressing plant-produced reactive oxygen species (Trivedi et al., 2020). Successful colonization of fungus can improve the adaptive of grassland plant response to grazing stress by enhancing nutrition acquisition (Xu et al., 2018). Interestingly, Xie et al. (2018) also confirmed that ABA treatment inhibited the accumulation of ROS by inducing the expression of enzymes such as SOD.

**Figure 6:**
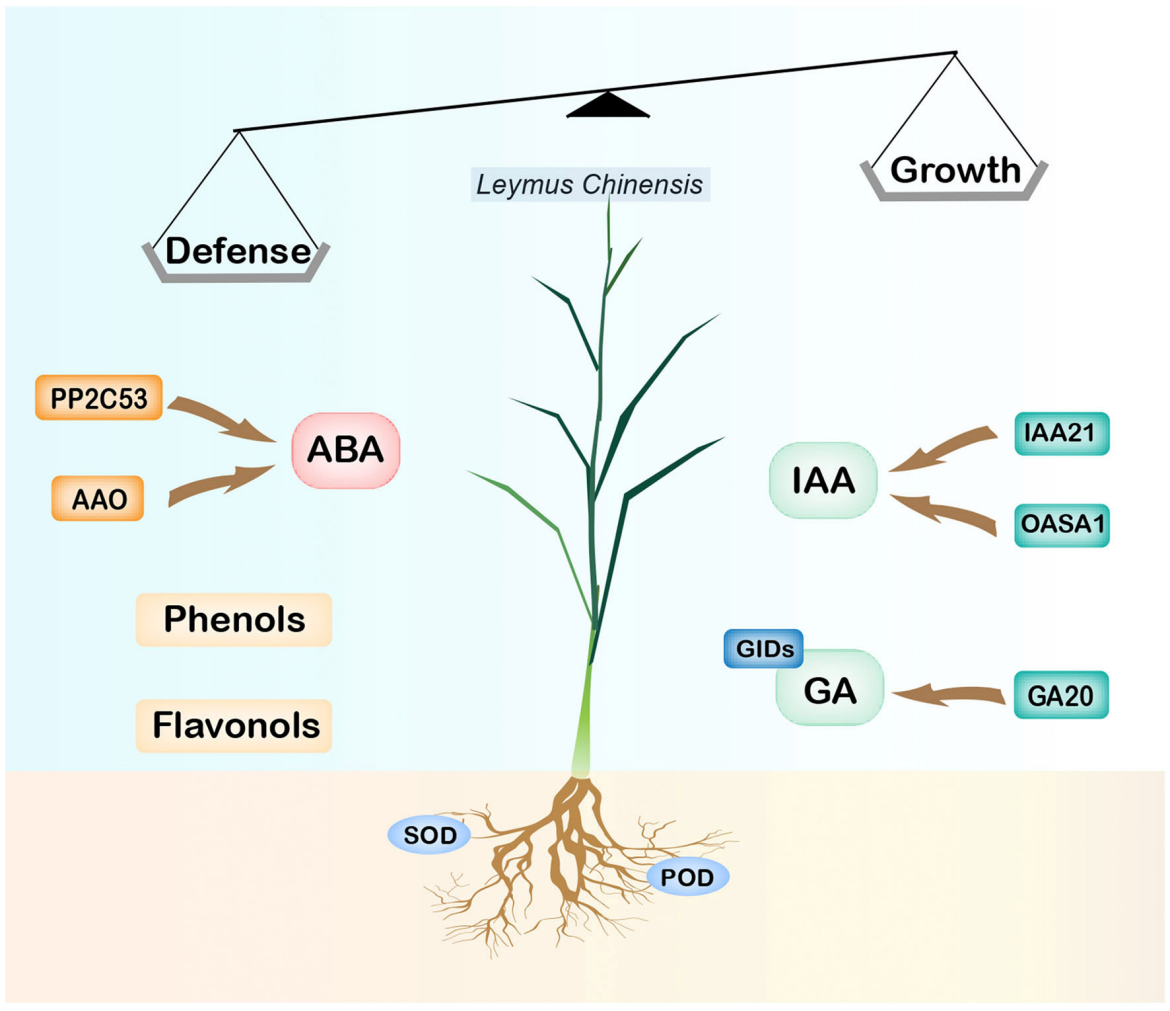
A model for the mechanisms under long-term OG stresses. For the downregulation of synthetic genes OASAs, G20, and receptor protein genes GIDs, the contents of IAA and GA are reduced, thus limiting plant growth; the increase of the ABA level induced individual defense and further inhibited plant growth. The contents of flavonoids and phenols in leaves increased, while the activities of superoxide dismutase (SOD) and peroxidase (POD) in roots decreased.

The most obvious finding to emerge from this research is that the stress memory of plants induced by long-term OG may be regulated by the elevated level of ABA and activation of defense weapons such as phenolics and flavonoids, as well as the inhibition of the biosynthesis of IAA and GA and their reception and signal transduction process (Figure 6). Moreover, the root tends to maintain growth by lowering its defense with decreased secondary metabolites and enzymes. These changes in phytohormones were modulated by several key genes such as *YUCs, OASAs, G20s, AAO*, and *NCEDs* which were key players in the biosynthesis of ABA, IAA, and GA. These findings will expand our knowledge of how grassland plants regulate their phenotype by phytohormones after experiencing long-term OG. Several questions remain unanswered at present. Does the situation change under different development stages? How many generations can the grazing-induced stress memory pass down? Further investigation is needed for us to establish a greater degree of accuracy on this matter, which could also be conducted to determine the effectiveness of the recovery procedure and mechanism of grassland degeneration.

## Data Availability Statement

Our data is publicly available by providing a valid accession number BioProject ID: PRJNA830275.

## Author Contributions

WR communicated with all authors as corresponding author. KQ, WR, YC, and YL contributed to conception and design of the study. KQ, KG, and JY prepared the experimental materials and performed the statistical analysis. KQ wrote the first draft of the manuscript. WR and EF read and revised the manuscript. All authors contributed to manuscript, read, and approved the submitted version.

## Funding

We are sincerely grateful for funding from the National Natural Science Foundation of China (Nos. 32060407 and 31872407), the National Natural Science Foundation of Inner Mongolia (No. 2020MS03029), and the Major Science and Technology Projects of Inner Mongolia (Nos. 2020GG0063, 2021ZD0008, and 2020ZD0020).

## Conflict of Interest

YL was employed by Inner Mongolia Mongolian Grass Seed Industry Science and Technology Research Institute Co., Ltd.. The remaining authors declare that the research was conducted in the absence of any commercial or financial relationships that could be construed as a potential conflict of interest.

## Publisher's Note

All claims expressed in this article are solely those of the authors and do not necessarily represent those of their affiliated organizations, or those of the publisher, the editors and the reviewers. Any product that may be evaluated in this article, or claim that may be made by its manufacturer, is not guaranteed or endorsed by the publisher.

## Supplementary Material

Supplementary Figure S1 Expression levels of auxin (IAA), gibberellic acid (GA), and abscisic acid (ABA)-related genes.

Supplementary Figure S2 The activity of peroxidase (POD) and superoxide dismutase (SOD) in roots and leaves in response to two conditions. ^***^*P* < 0.001; 0.001 < ^**^*P* < 0.05; ^*^*P* < 0.05.
