# Supplementary figures and images for: Growth-Defense Trade-Offs Induced by Long-term Overgrazing Could Act as a Stress Memory
Source: Front Plant Sci. 2022 Jun 2;13:917354. doi: 10.3389/fpls.2022.917354 (PMC9201768; doi:10.3389/fpls.2022.917354)

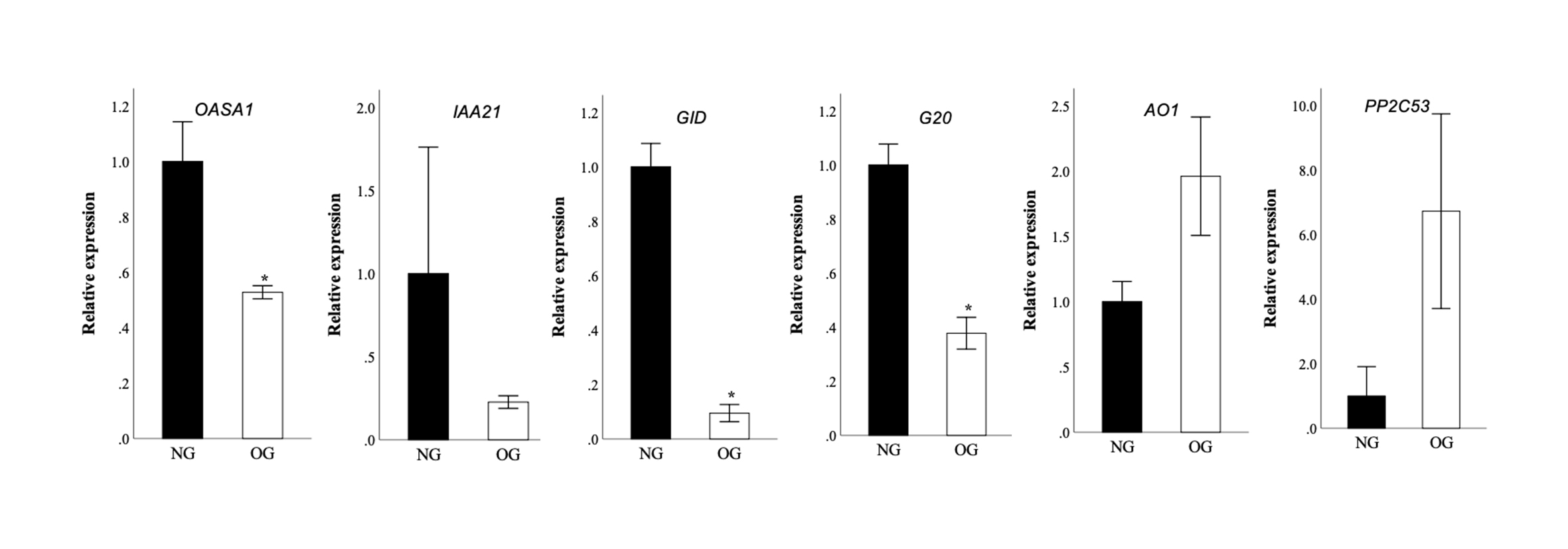

Supplement: Supplementary Figure S1 — Expression levels of auxin (IAA), gibberellic acid (GA), and abscisic acid (ABA)-related genes. [file Image_1.JPEG]

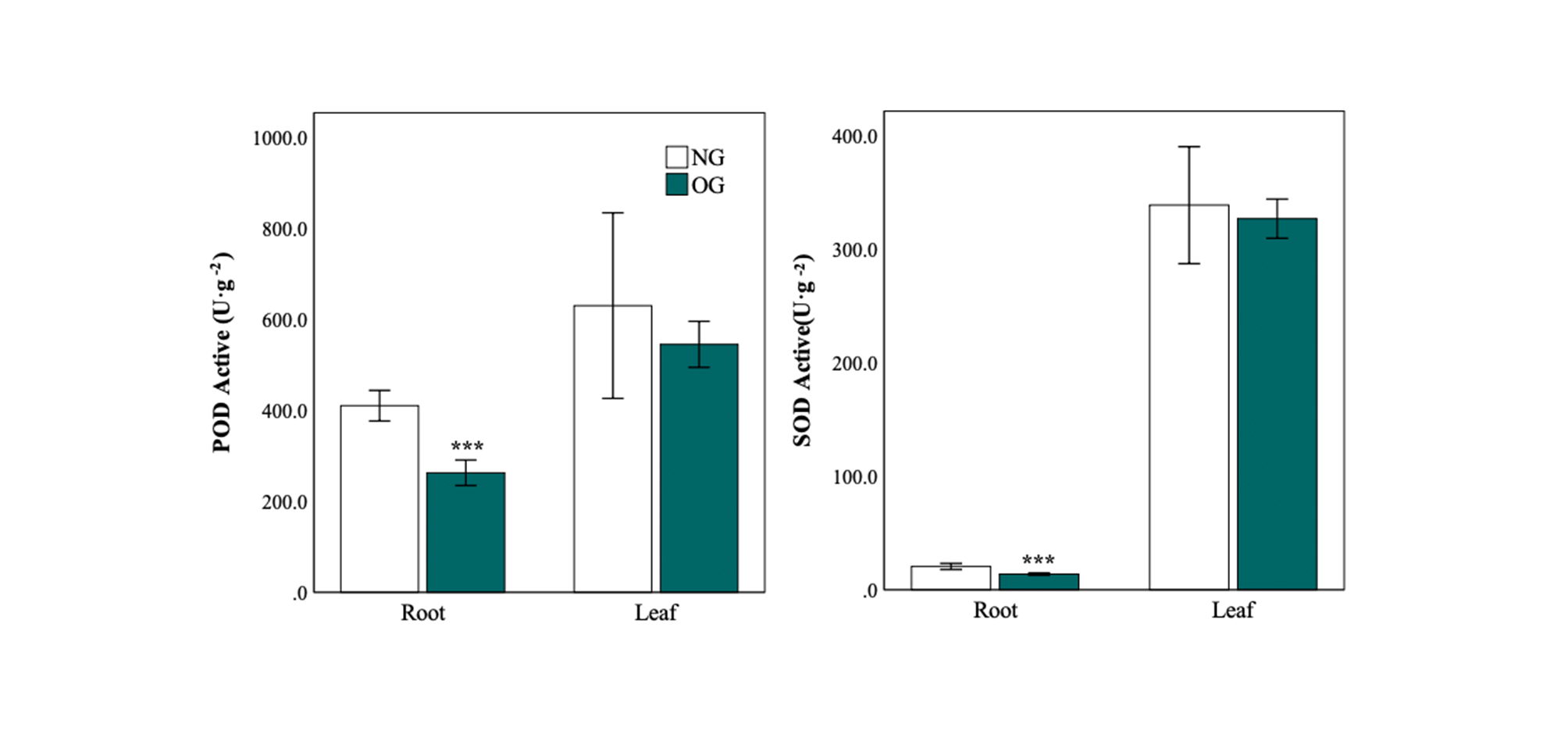

Supplement: Supplementary Figure S2 — The activity of peroxidase (POD) and superoxide dismutase (SOD) in roots and leaves in response to two conditions. ***P < 0.001; 0.001 < **P < 0.05; *P < 0.05. [file Image_2.JPEG]
