# Supplementary material for: Growth-Defense Trade-Offs Induced by Long-term Overgrazing Could Act as a Stress Memory
Source: Front Plant Sci. 2022 Jun 2;13:917354. doi: 10.3389/fpls.2022.917354 (PMC9201768; doi:10.3389/fpls.2022.917354)
Supplement: Supplementary file 3 [file Table_1.docx]

***Supplementary Material***

**Table S1 qRT-PCR primers of genes related to the biosynthesis and signal of IAA, GA and ABA in *Leymus chinensis* leaves**

| **Gene** | **Prime NO.** | **Primer Sequence（5'→3'）** | **Product size(bp)** |
| --- | --- | --- | --- |
| ***OASA1*** | **OASA1-2F** | ACCGAAACTCGAAGCGAGAA | **20** |
|  | **OASA1-2R** | CGAAGGAGTGAGCTTTGTTCC | **21** |
| ***IAA21*** | **IAA21-2F** | TGCCCTGCAATTTAGTAGGCAG | **22** |
|  | **IAA-2R** | ATTCGCCATAGCACAGGTTGA | **21** |
| ***GID*** | **GID-1F** | GAGAGCCAGCCTGAAAATTGG | **21** |
|  | **GID-2R** | CGTCTTTCTCCCGGCAAGTA | **20** |
| ***G20*** | **G20-1F** | CGCCACTGTACCCAGGTTAG | **20** |
|  | **G20-1R** | GAGAAGGTTGACCTGAGCCC | **20** |
| ***AO1*** | **AO1-1F** | ACTCAGACGGTCTCGTGGTA | **20** |
|  | **AO1-1R** | CCGAAGCTGTATCGAAACCAA | **21** |
| ***PP2C53*** | **PP2C53-1F** | ATGCTTTGATTGCCTTGGCG | **20** |
|  | **PP2C53-1R** | ACTAGCTGCCCTAAGCGTTG | **20** |
